# The Role of Corticosteroids in Treating Acute Ocular Toxoplasmosis in an Immunocompetent Patient: A Case Report

**DOI:** 10.3389/fmed.2022.843050

**Published:** 2022-06-29

**Authors:** Hung-Yi Lin, Wan-Ju Annabelle Lee

**Affiliations:** ^1^Medical Education Center, Chi Mei Medical Center, Tainan, Taiwan; ^2^Department of Ophthalmology, Chi Mei Medical Center, Tainan, Taiwan; ^3^Institute of Clinical Pharmacy and Pharmaceutical Sciences, College of Medicine, National Cheng Kung University, Tainan, Taiwan; ^4^Department of Optometry, Chung Hwa University of Medical Technology, Tainan, Taiwan

**Keywords:** ocular toxoplasmosis, corticosteroid, retinal necrosis, retinal detachment, choroidoretinitis

## Abstract

**Background:**

This study aimed to report a case who was treated with corticosteroids and anti- parasitic agents for ocular toxoplasmosis, but who progressed to acute retinal necrosis, and finally retinal detachment.

**Case Presentation:**

A 42-year-old man presented to the ophthalmology clinic with a 1-month history of progressive blurred vision and floaters in his right eye. His best visual acuity (VA) was 20/20 in both eyes. The anterior segment was unremarkable. Funduscopic examination of the right eye revealed active lesions of whitish foci of chorioretinitis with surrounding edema along the superonasal vessels, and retinal vasculitis with perivascular sheathing. Serologic testing was positive for *Toxoplasma gondii* IgM and IgG, but negative for other virus- and syphilis infections. Ocular toxoplasmosis was diagnosed. Corticosteroids and anti-parasitic agents were given simultaneously, but his right eye VA became 20/100. Funduscopic examination revealed retinal necrosis with localized retinal breaks. We immediately performed focal photocoagulation, however, his right eye progressed to retinal detachment and required vitrectomy.

**Conclusion:**

Early administration of systemic corticosteroids in patients with acquired acute ocular toxoplasmosis may lead to complications that impair vision. Intensive observation should be arranged after corticosteroid use.

## Introduction

Toxoplasmosis is an infection caused by the intracellular protozoan parasite *Toxoplasma gondii*. Primary infection in immunocompetent persons is usually asymptomatic and self-limited. The most common manifestations of acute toxoplasmosis in immunocompetent patients are cervical lymphadenopathy and flu-like symptoms. Toxoplasmosis may also present as ocular disease, which is the most frequent etiology of infectious posterior uveitis (1). In immune intact patients, typical clinical manifestations of ocular toxoplasmosis have been described as a nidus of fluffy, white, necrotizing retinitis or choroidoretinitis adjacent to a variably pigmented chorioretinal scar. Clinical diagnosis in most cases is based on typical presentation upon funduscopic examination. Serological analysis may be helpful in recently-acquired cases (2). In atypical cases, such as the absence of a retinochoroidal scar, analyzing antibody levels and parasitic DNA in the aqueous humor and serum specimen may be necessary to confirm the diagnosis (3). The current treatment regimen with anti-parasitic agents has been discussed widely without reaching consensus. Corticosteroid therapy is used as a part of the therapeutic regimen to regulate the immune response, however, this strategy may lead to damage to the ocular tissues. A combination of anti-parasitic agents and corticosteroids is suggested in most clinical scenarios, but the timing for commencement of corticosteroids has been subject to some controversy (4).

Here, we report an ocular toxoplasmosis case of an immunocompetent young male who presented with acute focal choroidoretinitis without the typical scarring lesion, and who progressed to localized retinal detachment soon after simultaneous use of anti-parasitic agents and corticosteroids.

## Case Presentation

**FIGURE 1:**
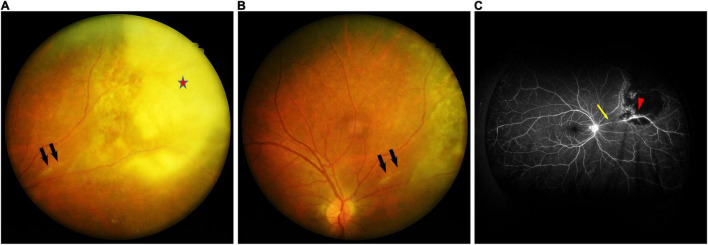
**(A)** Fundus photographs of the right eye showing whitish foci of chorioretinitis (red star) with surrounding edema along the superonasal vessels, and retinal vasculitis with perivascular sheathing (black arrow). **(B)** Fundus photographs of the right eye showing retinal vasculitis with perivascular sheathing (black arrow) at the same position as in panel **(A)**. **(C)** Fluorescein angiography of the right eye demonstrating obliteration of the blood flow with a non-perfusion area (yellow arrow) and leakage at the vessel wall (red arrowhead).

A 42-year-old man presented to the ophthalmology clinic with a 1-month history of progressive blurred vision and floaters in his right eye. He had no medical history. On ocular examination, the visual acuity (VA) was 20/20 in both eyes. The anterior segment was unremarkable. Funduscopic examination of the right eye revealed active lesions with whitish foci of choroidoretinitis with surrounding edema along the superonasal vessels, and retinal vasculitis with perivascular sheathing (Figures 1A,B). Serologic testing was positive for *Toxoplasma gondii* IgM and IgG, HSV-1 IgG, HSV-2 IgG, and CMV IgG, and negative for syphilis and Anti-HIV. Optical coherence tomography (OCT) of the macula revealed mild perifoveal edema in the right eye. Fluorescein angiography of the right eye showed obliteration of the blood flow with a non-perfusion area and leakage at the vessel wall (Figure 1C).

Based on the patient’s history, ophthalmic findings, and serology results, an infectious cause was highly suspected. We performed aqueous humor tapping for viral and tuberculosis polymerase chain reaction (PCR) testing to exclude possible causative agents. The PCR result showed no evidence of any viral or TB infection. We also performed a serum quantiferon gold test for the patient in case of recurrent tuberculosis, but the result was negative.

**FIGURE 2:**
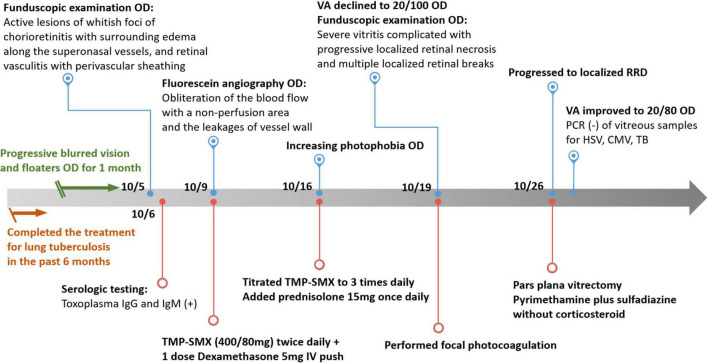
Timeline of patient’s presentation and treatment.

Thus, ocular toxoplasmosis was diagnosed. We consulted an infection specialist regarding the survey and management of potential systemic toxoplasmosis. The infection was initially managed by the infection specialist with sulfamethoxazole-trimethoprim (400/80 mg) twice per day, and one dose of dexamethasone 5 mg intravenously, on the first day. Five days later, the patient complained of significantly increasing photophobia in the lesioned eye, so we titrated sulfamethoxazole-trimethoprim (400/80 mg) to 3 times per day and added prednisolone 15 mg per day, simultaneously. Two days later, his right eye visual acuity (VA) declined to 20/100. Funduscopic examination revealed severe vitritis complicated with progressive localized retinal necrosis and multiple localized retinal breaks. Although we performed focal retinal photocoagulation immediately, he progressed to localized rhegmatogenous retinal detachment 5 days later. He then underwent a successful pars plana vitrectomy. During the surgery, we sent the vitreous samples for PCR to detect other possible pathogens, including HSV, CMV, and TB, but the results were negative. His vision improved to 20/80 after the surgery, and he has been treated with pyrimethamine plus sulfadiazine since then. The retina was attached but his VA remained at 20/80. Figure 2 showcases the timeline with relevant data from the period of care.

## Discussion

Ocular toxoplasmosis typically affects the posterior pole of a single eye and lesions may be single, multiple, or satellites adjacent to retinal pigmented scars. Active lesions present as grayish-white spots of retinal necrosis adjacent to choroiditis, in addition to vasculitis, hemorrhage, and/or vitritis. Healing occurs from the periphery toward the center of the lesion, with variable pigmentary changes. The retina is the primary site of ocular infection by *T. gondii*, but the choroid, vitreous, and anterior chamber may also be involved (5). Anterior uveitis is a common finding, with mutton-fat keratic precipitates, cells, flare, and posterior synechiae. Retinal vasculitis and associated inflammatory reactions may be the only ophthalmic signs during the early stages of acquired ocular toxoplasmosis. The later development of retinitis or scars in the same eye, consistent with ocular toxoplasmosis, suggests parasites as the cause of the initial inflammation. The involvement of the underlying choroid is termed “choroidoretinitis” and describes the clinical image. Ocular complications include choroidal neovascularization, cataract, glaucoma, optic nerve atrophy, and retinal detachment (6, 7).

The diagnosis of ocular toxoplasmosis is usually clinical. With regard to the active lesions, vitritis is mostly the first factor causing visual symptoms. A considerable vision deterioration may manifest due to macular involvement, whereas peripheral lesions may not have an obvious effect on vision. Choroidoretinitis is the most prevalent feature of active intraocular inflammation in patients with ocular toxoplasmosis. However, many cases may present with substantial clinical variations leading to diagnostic difficulties (3, 8–10).

In immunocompetent patients, the active lesions tend to heal automatically within 2–4 months, leaving an atrophic area (resolving from the periphery to the center) that gradually leads to a hyper-pigmented scar due to disruption of the retinal pigment epithelium (RPE). The presence of *T. gondii* IgG antibodies cannot confirm a diagnosis of eye infection, but IgG negativity generally rules it out. Ocular toxoplasmosis may be diagnosed pathologically by way of histology (identifying cysts in biopsies stained with hematoxylin and eosin), by immunohistochemistry (detecting monoclonal or polyclonal antibodies), or by polymerase chain reaction (PCR) (10–12).

We report on an immunocompetent patient with progressive blurry vision whose initial differential diagnoses included acute retinal necrosis, ocular tuberculosis, and ocular toxoplasmosis. Acute retinal necrosis, which is commonly caused by VZV, HSV-1, HSV-2, and rarely CMV, generally occurs in immunocompetent patients and may develop without a systemic prodrome. PCR testing of the vitreous or aqueous humor is sufficient for diagnosis, and the serology test is rarely helpful (13). Ocular tuberculosis, which usually follows hematogenous spread, should also be considered, especially if the patient has had previous tuberculous infection. Definitive diagnosis of tuberculosis needs cultures or DNA amplification from the involved tissue. However, culture or biopsy from the involved tissues in cases of ocular tuberculosis is unpractical, because aqueous and vitreous paracentesis commonly fails to produce positive bacterial culture results (14). Our patient denied any respiratory symptoms, and his recent pulmonary radiographic exam showed no suspected lesion. Recently-acquired ocular toxoplasmosis may present as focal choroidoretinitis without a visible chorioretinal scar (15). Some studies also report that the sensitivity of PCR ranges from 27 to 36% in patients diagnosed with ocular toxoplasmosis (11). Since there were no positive results from either aqueous or vitreous samples, we made our diagnosis based on the ocular findings and the positive anti-toxoplasma IgM titer.

The treatment and prophylaxis of active ocular toxoplasmosis have been widely discussed. Typically, toxoplasmic choroidoretinitis in immunocompetent patients is expected to resolve within 1–2 months, since most infections with active ocular toxoplasmosis are thought to be self-limiting over the course of 1–2 months. Taking into account the benign natural course and the possibility of toxicity from anti-parasitic drugs, the therapeutic approach for each individual with active infection should seek to avoid unnecessarily high rates of drug-induced morbidity. Anti-parasitic agents are suggested for acquired immunocompetent patients with active toxoplasmic choroidoretinitis whenever the optic disc or fovea is involved, or if there is severe vitritis or vasculitis, giant diameter size, multiple active lesions, and prolonged clinical course (16). Treatment is also warranted for patients with atypical presentations (17). Current therapeutic strategy for ocular toxoplasmosis aims to stop the parasite reproducing and to eradicate it, while suppressing inflammation to control tissue damage from the immune response. The anti-parasitic agents pyrimethamine and sulfadiazine plus corticosteroid form the classical triple-treatment for ocular toxoplasmosis (17). Trimethoprim-sulfamethoxazole has been put forward as another therapeutic option because of lower cost, comparable efficacy, and safety for the prophylaxis of recurrent toxoplasmic choroidoretinitis (18). One currently used approach (except in individuals who are allergic to sulphonamides) involves systemic treatment with a combination of trimethoprim (800 mg) and sulfamethoxazole (160 mg), administered twice daily for 6 weeks, which is generally well-tolerated. This strategy is equivalent to the standard daily treatment with pyrimethamine (50 mg) in combination with sulfadiazone (1000 mg) 4 times a day and folinic acid (15 mg) twice weekly (to prevent anemia), for 6 weeks (19). Corticosteroids were thought to be beneficial for patients by suppressing the intraocular inflammation (16). However, corticosteroid monotherapy has been reported to worsen the clinical course and cause serious complications (15). Despite several published articles proposing various dosages of adjunctive corticosteroids, there is still no strong evidence from randomized controlled trials to support this adjuvant therapy being helpful for patients with ocular toxoplasmosis, especially as regards improvement of their vision (20, 21). The combined antibiotic and steroid approach should be limited to patients with an exaggerated inflammatory response, since it remains unclear whether supplementary treatment with corticosteroids does indeed effect an improvement in the outcome over and above that achieved with anti-parasitic agents alone.

This case is noteworthy because it shows how, even in an immunocompetent patient under proper management with anti-parasitic agents accompanied with corticosteroids, the severe inflammation still led to retinal detachment and impaired vision. Corticosteroids might minimize the inflammatory damage in most cases, but the route, dosage, and timing of administration are still subject to controversy. Early administration of corticosteroids together with the anti-parasitic agents, as in our case, might not be beneficial but might actually lead to advanced complications in the ocular tissues. We should keep in mind that iatrogenic immunosuppression by corticosteroids may cause aggressive choroidoretinitis and further complications such as retinal detachment. Detailed ophthalmic examinations are required to prevent future vision loss.

## Data Availability Statement

The original contributions presented in the study are included in the article/supplementary material, further inquiries can be directed to the corresponding author.

## Ethics Statement

Ethical review and approval was not required for the study on human participants in accordance with the local legislation and institutional requirements. The patients/participants provided their written informed consent to participate in this study.

## Author Contributions

H-YL contributed to writing the original manuscript. Both authors contributed to the literature research and preparation of the manuscript and figures, were responsible for the design of the case report, and read and approved the final manuscript.

## Conflict of Interest

The authors declare that the research was conducted in the absence of any commercial or financial relationships that could be construed as a potential conflict of interest.

## Publisher’s Note

All claims expressed in this article are solely those of the authors and do not necessarily represent those of their affiliated organizations, or those of the publisher, the editors and the reviewers. Any product that may be evaluated in this article, or claim that may be made by its manufacturer, is not guaranteed or endorsed by the publisher.

## Abbreviations

PCR: polymerase chain reaction
DNA: deoxyribonucleic acid
VA: visual acuity
OCT: optical coherence tomography
HSV: herpes simplex virus
CMV: cytomegalovirus
TB: tuberculosis
M. tuberculosis: mycobacterium tuberculosis
VZV: varicella-zoster virus
IgG: immunoglobulin G
IgM: immunoglobulin M.
